# Comparative study of multiple approaches for identifying cultivable microalgae population diversity from freshwater samples

**DOI:** 10.1371/journal.pone.0285913

**Published:** 2023-07-07

**Authors:** Amal A. Badr, Walid M. Fouad

**Affiliations:** 1 Biotechnology Graduate Program, School of Sciences and Engineering, The American University in Cairo, New Cairo, Egypt; 2 Department of Biology, School of Sciences and Engineering, The American University in Cairo, New Cairo, Egypt; National Cheng Kung University, TAIWAN

## Abstract

The vast diversity of microalgae imposes the challenge of identifying them through the most common and economical identification method, morphological identification, or through using the more recent molecular-level identification tools. Here we report an approach combining enrichment and metagenomic molecular techniques to enhance microalgae identification and identify microalgae diversity from environmental water samples. From this perspective, we aimed to identify the most suitable culturing media and molecular approach (using different primer sets and reference databases) for detecting microalgae diversity. Using this approach, we have analyzed three water samples collected from the River Nile on several enrichment media. A total of 37 microalgae were identified morphologically to the genus level. While sequencing the three-primer sets (16S rRNA V1-V3 and V4-V5 and 18S rRNA V4 region) and aligning them to three reference databases (GG, SILVA, and PR^2^), a total of 87 microalgae were identified to the genus level. The highest eukaryotic microalgae diversity was identified using the 18S rRNA V4 region and alignment to the SILVA database (43 genera). The two 16S rRNA regions sequenced added to the eukaryotic microalgae identification, 26 eukaryotic microalgae. Cyanobacteria were identified through the two sequenced 16S rRNA regions. Alignment to the SILVA database served to identify 14 cyanobacteria to the genera level, followed by Greengenes, 11 cyanobacteria genera. Our multiple-media, primer, and reference database approach revealed a high microalgae diversity that would have been overlooked if a single approach had been used over the other.

## Introduction

The advancement of next-generation sequencing (NGS) methods has made significant progress in microbiome research. Nevertheless, the gap in identifying the unknown content and “unculturable” communities and the necessity of the presence of live biological matter (the cultured microorganism) as a proof-of-concept test has allowed the culture methods to resurface. The utilization of the traditional microscopy techniques for directly studying of the environmental samples is subjective with the possibility of either overestimation or underestimation of diversity if used as a sole identification method. Traditional microscopy techniques in microalgae identification depend on the level of experience of the person conducting it where small-sized cells are prone to either be completely missed or underrepresented [1]. Culturing samples before metagenomics has been utilized in various studies investigating the microbiota in different ecosystems; this approach is widely known as culturomics. Culturomics has been used for microbiome analysis of humans [2–8], marine sediments [9, 10], soil [11], root samples [12], and mouse gut [13]. Hence, we decided to utilize this approach in identifying microalgae diversity from freshwater environmental samples.

One of the approaches to identifying microalgae from environmental samples is the traditional morphological identification method, as mentioned above, through enrichment of the environmental sample before microscopy identification. The commonly used media for microalgae culture in the literature includes Blue-Green media (BG-11) and Bold’s Basal media (BBM), which have been widely used to identify and maintain species diversity as they can sustain a wide range of species [14].

Microalgae identification can also be made through the molecular level analysis of conserved regions of the DNA, such as Ribulose Bisphosphate Carboxylase Large subunit gene (rbcL), 16S and 18S ribosomal RNA (rRNA) genes used for the profiling of prokaryotic and eukaryotic microalgae [15–17]. The reference database to align the sequenced results is equally important to the DNA barcodes sequenced. Hence, it is crucial to identify the most suitable database for microalgae identification. The SILVA database is predominantly used and is based on the small subunit (SSU) rRNAs that include the 16S and 18S rRNA in prokaryotes and eukaryotes, respectively. Therefore, they cover the three domains of life: Bacteria, Archaea and Eukarya. The SILVA database can assign taxonomic ranks to the genus level [18]. Greengenes (GG) database constitutes bacterial/archaeal 16S rRNA gene. The GG database is constructed from public aligned and checked databases for chimera [19, 20]. At the same time, the Protist Ribosomal Reference (PR^2^) database contains sequences of the SSU rRNA and rDNA of the kingdom Eukaryota. The PR^2^ database identifies eukaryotes through nuclear-encoded sequences, the 16S rRNA, present in the mitochondria or plastid of eukaryotic organisms [21]. Both GG and PR^2^ provide taxonomic annotation down to the species level.

The influence of enrichment media on microalgae population growth and diversity in the literature remains elusive; however, still utilized [17, 22–24]. We previously investigated the efficiency of the commonly used BG-11 and BBM in microalgae enrichment for diversity identification and the effect of vitamin enrichment and reduced media nutrient on diversity enrichment. The results obtained using the reduced media provided insight into the limitation of both media and the need to investigate other media compositions [25]. Hence, this study aims to improve the detection and quantification of microalgae by comparing the usage of different enrichment media, DNA barcodes, and reference databases in the identification of cultivable microalgae diversity from an environmental sample. Our specific aims are: (1) increasing algal abundance above the detection threshold using four different enrichment media and (2) the utilization of multi-primer sets for SSU rRNAs (16S and 18S rRNA) and; (3) using different reference databases (SILVA, Greengenes (GG), and Protist Ribosomal Reference database (PR^2^)) to evaluate their competence in the identification of microalgae.

## Materials and methods

### Study areas and sample enrichment

To investigate the competence of the different databases and the multi-primers studied, three water samples were collected from the Nile River in the Cairo governate (no specific permissions were required for sampling the three locations studied, which complied with all relevant regulations.) **(S1 Table)** and enriched with different nutrient media. Water samples were collected 20 cm below the water surface from each location on the same day and kept in the fridge until transported and processed in the lab the next day. Portions of each sample were used for physicochemical analysis, and the remaining water samples were used to inoculate the nutrient media. Four different nutrient media were used for algal enrichment: Blue-Green medium (BG-11), Bold’s Basal medium (BBM), half-strength Murashige and Skoog medium (MS), and a Modified medium (MM) that fits in the middle of the BG-11 and BBM in terms of the nitrogen and phosphorus composition **(S2 Table).** Each volume of nutrient media was inoculated with one-tenth of the environmental sample. For all tested factors, cultures were carried out in triplicate with continuous aeration and maintained for three weeks in a JSR-Growth Chamber 3-Side Illumination (model JSPC-960C2) at a temperature of 22±1°C. The light/dark regime was adjusted to 12h:12h, at a light intensity of 28 μmol photons m^−2^ s^−1^ [26]. Microalgae population growth was monitored by performing a cell count every four days using a Neubauer Improved Hemocytometer counting chamber for each replica under Zeiss AxioStar Plus light microscope at 400x magnification based on [27, 28] **(S1 Fig).**

### Physicochemical analysis

The physicochemical analysis was performed to assess the composition of the microalgae’s natural habitat identified in each location’s water samples (**S3 Table**). The physical and chemical characteristics measured, including salinity, pH, and electrical conductivity (EC), were measured using a multimeter-probe (330i, WTW, Germany). The presence of cations and anions, such as sodium, chloride, sulfate, potassium, and calcium, were measured according to standard methods [29]. Total ammonia, nitrate, and phosphorus levels were evaluated according to a standard protocol [30].

### Morphological identification of microalgae

Microalgae identification was based on the morphological characteristics observed under a bright-field microscope following the basic identification keys from references [31, 32]. The strains were examined under a light microscope (Leica) using the software LAS EZ (Leica DM500). Microalgae was identified at the genus level every seven days until the experiment was concluded.

### DNA extraction and sequencing

Total genomic DNA (gDNA) was extracted using the PowerSoil DNA Isolation kit (MoBio, Carlsbad, CA) following the manufacturer’s instruction with a minor alteration. Briefly, after 14 days of media enrichments, the cultured algae were collected from 200ml of each sample by centrifugation at 4000 rpm for 30 minutes. About 150–250 mg of the wet pellet was then snap-frozen by dropping the Eppendorf containing the wet pellet in liquid nitrogen. The frozen algae mass was ground using a pestle and used for gDNA extraction using the PowerSoil DNA Isolation kit following the manufacturer’s instructions. The concentration and purity of the DNA extracted were confirmed using LVis Plate SPECTROstar® Nano (BMG LABTECH, UK), and integrity was confirmed using 1% agarose gel.

Three primer sets hyper-variable regions of 16S rRNA V1-V3 [33, 34], V4-V5 [33] and 18S rRNA V4 [35, 36] were used for sequencing (**S4 Table**). PCR and sequencing was performed at the Beijing Genomics Institute (BGI) (BGI-Tech, BGI-Shenzhen, Shenzhen, China). PCR amplification was performed using 2× Phanta Max Master kit (Vazyme Biotech Co., China) using 30ng gDNA as template. PCR conditions were as follows: initial denaturation at 94°C for 3 min; 30 cycles of 94°C for 30 s, annealing for 30s and the annealing temperature for each primer set was 50°C (16S V1-V3) and 53°C (16S V4-V5 and 18S V4), extension at 72°C for 45 s; and a final extension at 72°C for 10 min. The PCR products were purified using AMPure XP beads, and the purified product was used to construct the DNA library. The prepared DNA library was used for sequencing. The sequencing was performed at the Beijing Genomics Institute (BGI) (BGI-Tech, BGI-Shenzhen, Shenzhen, China) using Illumina HiSeq 2500 platform to generate 250/300bp paired-end (PE) reads.

### Raw sequence processing and data analysis

Trimmed fastq files were filtered using MOTHUR v1.42.6 following the pipeline from the MiSeq standard operating procedure (SOP) available on their website (www.mothur.org/wiki/MiSeq_SOP) [37]. Briefly, the fastq files were converted to fasta, and contigs were assembled using make.contigs. The files were screened using the MOTHUR command screen.seq. Reads selected were 457, 378, and 305bp fromthe 16S rDNA, V1-V3, V4-V5, and 18S rDNA sequences, respectively. Sequences with homopolymers longer than 8bp were discarded. The remaining sequences were de-replicated (unique.seqs) to merge duplicates and reduce the number of sequences to analyze. The remaining sequences were aligned to a database. The sequences of the 16S rDNA were aligned to the Greengenes database (Gg_13_8_99) and Protist Ribosomal Reference database (PR^2^) [21] 18S rDNA was aligned to the SILVA database (Silva.nr_v132). The lowest level of the taxonomic hierarchy present in SILVA is the genus, while in Greengenes lowest taxonomic level that can be identified is the species level [18, 38]. Chimeras in the sequences were also filtered and removed (chimera.uchime and remove.seqs). Sequences were split into groups based on their taxonomy at the order level, and operational taxonomic units (OTUs) were assigned using the dist.seqs command. Alpha diversity were calculated based on normalized OTU abundance information obtained using the sample with the fewest sequences as a standard. The diversity indices calculated include Good’s coverage, species observed (S_obs_), Shannon’s diversity index, Simpson’s diversity index (InvSimpson), and Berger-Parker, and they were calculated using the MOTHUR SOP [39]. Non-metric multidimensional scaling (NMDS) was conducted with the Bray-Curtis index distance method for beta diversity index.The molecular data were further analyzed and visualized with R 3.6.1 packages (“vegan”, “phyloseq”, “taxa”, and “ggplot2”) executed via RStudio (“Open source and enterprise-ready professional software for data science-RStudio”; “R: The R Project for Statistical Computing”).

## Results

### Overview of the freshwater microalgae taxa identified across different nutrient media

Pursuing the possibility of improving the media to better enrich the microalgae population and provide an evenness to the present diversity and enable the enrichment of rare species in the community. The growth rate of the algal population on the four different media tested indicated a comparable growth rate throughout the study. None of the media hindered the population growth; hence all were suitable for microalgae enrichment **(S1 Fig).**

**Fig 1:**
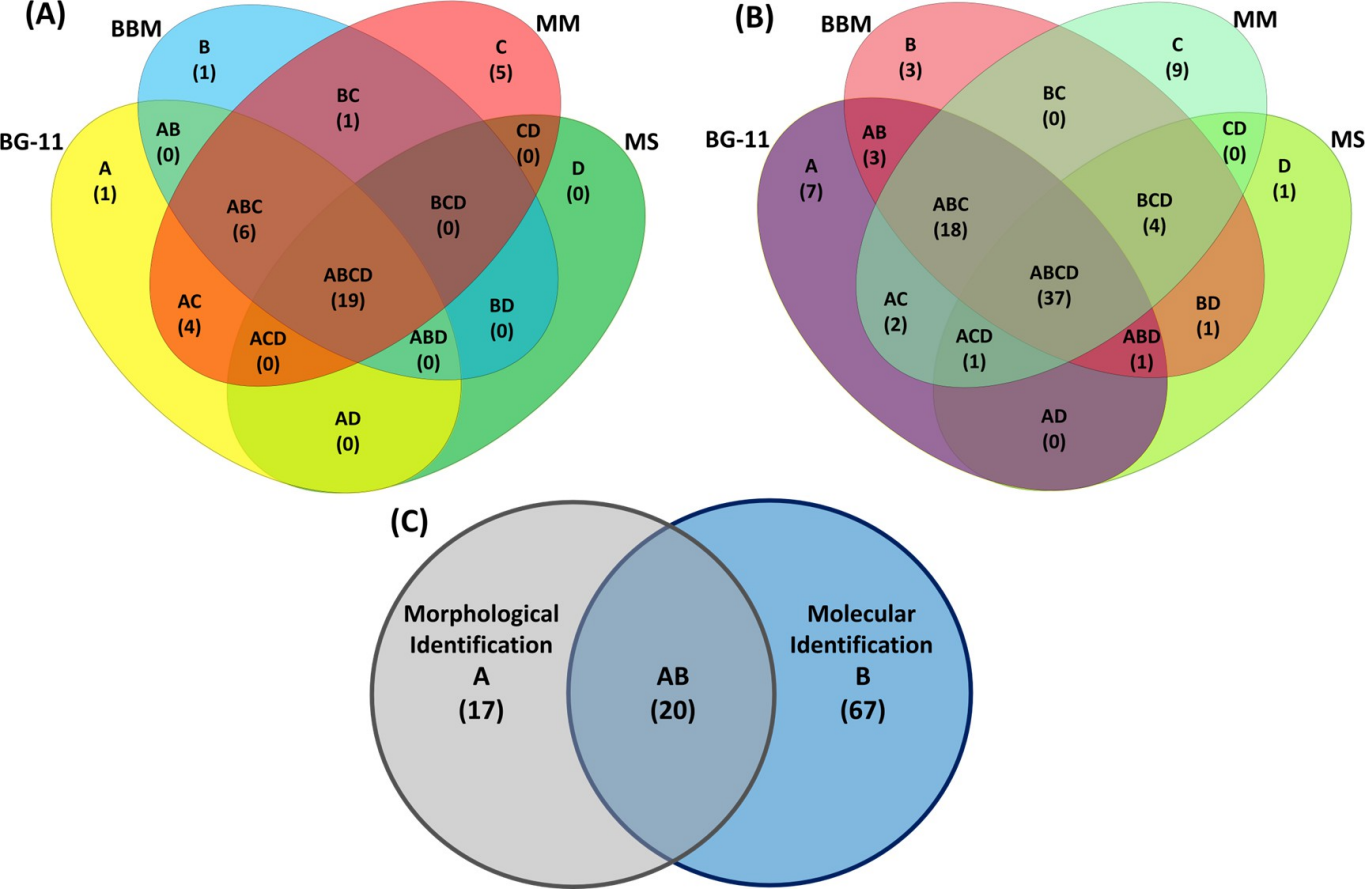
Freshwater microalgae taxa identified across different nutrient media. Venn diagram illustrating the number of shared and unique freshwater microalgae taxa across different culture media. The number of microalgae taxa identified on media BG-11, BBM, MM, and MS using two identification methods (A) Morphological identification, (B) Molecular-level identification using different DNA barcodes. (C) The total number of microalgae taxa commonly shared or uniquely identified using morphological and molecular identification methods. The genera identified are presented in **S5 Table**.

The morphological assessment of the samples using the four different nutrient media aided in identifying 37 microalgae to the genus level. The highest relative abundance belonged to *Chlorella*, *Scenedesmus*, *Ankistrodesmus*, and *Selenastrum*. The highest diversity was identified when the sample was cultured on MM (36 genera), followed by BG-11 (31 genera), BBM (26 genera), and MS media (20 genera) (**Fig 1A, S5 Table**). The half-strength MS media had the lowest diversity, and all the genera identified through the MS media were identified with the other media. We decided to continue molecular analysis, including the MS medium, because there could be several limitations to morphological analysis and that includes, the volume sampled, the number of microscopic fields visualized from a single sample, and the possibility of human error. As the highest algal diversity was observed after 14 days, DNA extraction was conducted on all samples on day 14 [25]. Therefore, the DNA extraction for the molecular level analysis was conducted on the samples on the 14th day.

Using the three primer sets and sequencing through the Illumina HiSeq platform, the three databases identified 87 microalgae to the genus level. Similar to the results of the morphological analysis, the highest diversity was identified using the MM media (71 genera), followed by BG-11 (69 genera), BBM (67 genera), and MS media (45 genera) (**Fig 1B**). However, *Raphidonema* was only identified on half-strength MS media through molecular analysis.

By employing morphological and molecular methods, the sample enrichment media successfully aided in the identification of 104 microalgae at the genus level (**Fig 1C** and **Table 1**). The highest diversity was identified through the sequencing using the three primer sets sequencing sections of the hypervariable regions of 16S and 18S rRNA genes when compared to the morphological level identification. Seventeen genera were uniquely identified through the morphological level analysis, while the molecular-based approach identified 67 unique genera (**Fig 1C**).

**Table 1:** List of the algal genera identified in this study through both morphological and molecular- level.

Genus^Location^* (By Phyla)	Enrichment media*	Identification Method							
		Morphology	SILVA			GG		PR^2^	
			18S V4	16S V1-V3	16S V4-V5	16S V1-V3	16S V4-V5	16S V1-V3	16S V4-V5
**Bacillariophyta **									
***Amphora*** ^***3***^	***A***	**-**	**-**	**-**	**-**	**-**	**-**	**+**	**-**
***Araphid pennate*** ^***1*,*2*,*3***^	***A B C D***	**-**	**-**	**-**	**-**	**-**	**-**	**-**	**+**
***Aulacoseira*** ^***1*,*2*,*3***^	***A B C***	**-**	**+**	**-**	**-**	**-**	**-**	**+**	**-**
***Cyclotella*** ^***1*,*2*,*3***^	***A B C D***	**+**	**+**	**-**	**-**	**-**	**-**	**+**	**-**
***Discostella*** ^***1*,*3***^	***A B C***	**-**	**+**	**-**	**-**	**-**	**-**	**-**	**-**
***Fistulifera*** ^***2***^	***A***	**-**	**-**	**-**	**-**	**-**	**-**	**+**	**-**
***Florenciella*** ^***2*,*3***^	***B C***	**-**	**-**	**-**	**-**	**-**	**-**	**+**	**-**
***Fragilaria*** ^***1*,*2***^	***A C D***	**-**	**-**	**-**	**-**	**-**	**-**	**+**	**-**
***Nannochloropsis*** ^***1*,*2*,*3***^	***A B C***	**-**	**-**	**-**	**-**	**-**	**-**	**-**	**+**
***Nitzschia*** ^***1*,*2*,*3***^	***A B C D***	**+**	**+**	**-**	**-**	**-**	**-**	**+**	**+**
***Ochromonas*** ^***1*,*2*,*3***^	***A B C D***	**-**	**+**	**-**	**-**	**-**	**-**	**-**	**+**
***Pedospumella*** ^***1*,*2*,*3***^	***A B C D***	**-**	**+**	**-**	**-**	**-**	**-**	**-**	**-**
***Polar Centric*** ^***1*,*2*,*3***^	***A B C D***	**-**	**-**	**-**	**-**	**-**	**-**	**+**	**+**
***Poterioochromonas*** ^***1*,*2***^	***B C D***	**-**	**+**	**-**	**-**	**-**	**-**	**-**	**-**
***Raphid pennate*** ^***1*,*2*,*3***^	***A B C D***	**-**	**-**	**-**	**-**	**-**	**-**	**+**	**+**
***Spumella*** ^***1*,*2*,*3***^	***A B C***	**-**	**+**	**-**	**-**	**-**	**-**	**-**	**-**
***Staurosira*** ^***2***^	***C***	**-**	**+**	**-**	**-**	**-**	**-**	**-**	**-**
***Stephanodiscus*** ^***2*,*3***^	***C***	**-**	**+**	**-**	**-**	**-**	**-**	**-**	**-**
***Thalassiosira*** ^***1*,*2*,*3***^	***A B C***	**-**	**+**	**-**	**-**	**-**	**-**	**+**	**-**
***Ulnaria*** ^***1*,*2*,*3***^	***A B C D***	**-**	**+**	**-**	**-**	**-**	**-**	**+**	**-**
**Charophyta/Streptophyta **									
***Chlorokybus*** ^***1*,*2***^	***A B C***	**-**	**-**	**-**	**-**	**-**	**-**	**+**	**+**
***Cosmarium*** ^***2***^	***A***	**+**	**-**	**-**	**-**	**-**	**-**	**+**	**-**
***Gonatozygon*** ^***2***^	***B***	**-**	**+**	**-**	**-**	**-**	**-**	**-**	**-**
***Mougeotia*** ^***1***^	***B***	**+**	**-**	**-**	**-**	**-**	**-**	**-**	**-**
***Staurastrum*** ^***1*,*2*,*3***^	***A B C***	**+**	**+**	**-**	**-**	**-**	**-**	**+**	**-**
***Staurodesmus*** ^***3***^	***B***	**-**	**+**	**-**	**-**	**-**	**-**	**-**	**-**
**Chlorophyta **									
***Acutodesmus*** ^***1*,*2*,*3***^	***A B C D***	**-**	**-**	**-**	**-**	**+**	**+**	**+**	**+**
***Anctinastrum*** ^***1*,*2*,*3***^	***A B C D***	**+**	**-**	**-**	**-**	**-**	**-**	**-**	**-**
***Ankistrodesmus*** ^***1*,*2*,*3***^	***A B C D***	**+**	**+**	**-**	**-**	**-**	**-**	**-**	**-**
***Asterococcus*** ^***1*,*2***^	***C***	**-**	**+**	**-**	**-**	**-**	**-**	**-**	**-**
***Botryococcus*** ^***2*,*3***^	***B***	**+**	**-**	**-**	**-**	**-**	**-**	**-**	**-**
***Carteria*** ^***1*,*2*,*3***^	***A B C D***	**-**	**+**	**-**	**-**	**-**	**-**	**-**	**-**
***Chlamydamonas*** ^***1*,*2*,*3***^	***A B C D***	**+**	**+**	**-**	**-**	**+**	**+**	**+**	**+**
***Chlorella*** ^***1*,*2*,*3***^	***A B C D***	**+**	**+**	**-**	**-**	**-**	**-**	**+**	**+**
***Chlorochytrium*** ^***2*,*3***^	***B C***	**-**	**+**	**-**	**-**	**-**	**-**	**-**	**-**
***Choricystis*** ^***1*,*2*,*3***^	***A B C D***	**-**	**+**	**-**	**-**	**-**	**-**	**-**	**-**
***Chlorococcum*** ^***3***^	***C***	**+**	**-**	**-**	**-**	**-**	**-**	**-**	**-**
***Closteriopsis*** ^***1*,*2*,*3***^	***A B C D***	**-**	**-**	**-**	**-**	**-**	**-**	**+**	**+**
***Coelastrum*** ^***1*,*2*,*3***^	***A B C D***	**+**	**+**	**-**	**-**	**-**	**-**	**-**	**-**
***Crucigenia*** ^***1*,*2*,*3***^	***A B C D***	**+**	**-**	**-**	**-**	**-**	**-**	**-**	**-**
***Desmodesmus*** ^***1*,*2*,*3***^	***A B C D***	**-**	**+**	**-**	**-**	**-**	**-**	**-**	**-**
***Dictyosphaerium*** ^***1*,*2*,*3***^	***A B C D***	**+**	**-**	**-**	**-**	**-**	**-**	**-**	**-**
***Dunaliella*** ^***1*,*2*,*3***^	***A B C***	**-**	**-**	**-**	**-**	**+**	**-**	**+**	**-**
***Golenkinia*** ^***1*,*2*,*3***^	***A C***	**+**	**+**	**-**	**-**	**-**	**-**	**-**	**-**
***Gonium*** ^***1*,*2*,*3***^	***A B C D***	**+**	**-**	**-**	**-**	**-**	**-**	**+**	**+**
***Hydrodictyon*** ^***1*,*2*,*3***^	***A B C D***	**-**	**+**	**-**	**-**	**+**	**+**	**+**	**+**
***Koliella*** ^***1*,*2*,*3***^	***A B C D***	**-**	**-**	**-**	**-**	**-**	**-**	**-**	**+**
***Lagerheimia*** ^***1*,*2*,*3***^	***A B C D***	**+**	**-**	**-**	**-**	**-**	**-**	**-**	**-**
***Micractinium*** ^***1*,*2*,*3***^	***A B C D***	**+**	**+**	**-**	**-**	**-**	**-**	**-**	**-**
***Microglena*** ^***1*,*2*,*3***^	***A B C D***	**-**	**+**	**-**	**-**	**-**	**-**	**-**	**-**
***Monoraphidium*** ^***1*,*2*,*3***^	***A B C D***	**-**	**+**	**-**	**-**	**-**	**-**	**-**	**-**
***Mychonastes*** ^***1*,*2*,*3***^	***A B C***	**-**	**+**	**-**	**-**	**-**	**-**	**-**	**-**
***Neochlorosarcina*** ^***1*,*2*,*3***^	***B C D***	**-**	**+**	**-**	**-**	**-**	**-**	**-**	**-**
***Neodesmus*** ^***1*,*2***^	***A B C D***	**-**	**+**	**-**	**-**	**-**	**-**	**-**	**-**
***Oedogonium*** ^***1*,*2***^	***A B***	**-**	**-**	**-**	**-**	**-**	**-**	**+**	**+**
***Oocystis*** ^***1*,*2*,*3***^	***A B C D***	**+**	**+**	**-**	**-**	**-**	**-**	**+**	**+**
***Oophila*** ^***1*,*2*,*3***^	***A B C D***	**-**	**-**	**-**	**-**	**-**	**+**	**-**	**+**
***Parachlorella*** ^***1*,*2*,*3***^	***A B C D***	**-**	**-**	**-**	**-**	**-**	**-**	**+**	**-**
***Pectodictyon*** ^***2***^	***A***	**-**	**+**	**-**	**-**	**-**	**-**	**-**	**-**
***Pediastrum*** ^***1*,*3***^	***A B C***	**+**	**-**	**-**	**-**	**-**	**-**	**-**	**-**
***Pedinomonas*** ^***1*,*2*,*3***^	***A B C***	**-**	**+**	**-**	**-**	**-**	**-**	**-**	**-**
***Picochlorum*** ^***1*,*2*,*3***^	***A B C D***	**-**	**+**	**-**	**-**	**-**	**-**	**-**	**-**
***Pleodorina*** ^***1*,*2*,*3***^	***B C D***	**-**	**-**	**-**	**-**	**-**	**-**	**+**	**-**
***Raphidonema*** ^***1***^	***D***	**-**	**-**	**-**	**-**	**-**	**-**	**-**	**+**
***Scenedesmus*** ^***1*,*2*,*3***^	***A B C D***	**+**	**+**	**-**	**-**	**-**	**-**	**-**	**-**
***Selenastrum*** ^***1*,*2*,*3***^	***A B C D***	**+**	**-**	**-**	**-**	**-**	**-**	**-**	**-**
***Sphaeroplea*** ^***1*,*2*,*3***^	***A B C D***	**-**	**+**	**-**	**-**	**-**	**-**	**-**	**-**
***Tetracystis*** ^***1*,*2*,*3***^	***A B D***	**-**	**+**	**-**	**-**	**-**	**-**	**-**	**-**
***Tetradesmus*** ^***1*,*2*,*3***^	***A B C D***	**-**	**+**	**-**	**-**	**-**	**-**	**-**	**-**
***Tetraedron*** ^***1*,*2*,*3***^	***A B C D***	**+**	**-**	**-**	**-**	**-**	**-**	**-**	**-**
***Tetranephris*** ^***2***^	***C***	**-**	**+**	**-**	**-**	**-**	**-**	**-**	**-**
***Tetraselmis*** ^***1*,*3***^	***A B D***	**+**	**+**	**-**	**-**	**-**	**-**	**-**	**-**
***Tetraspora*** ^***1*,*2*,*3***^	***A B C D***	**+**	**-**	**-**	**-**	**-**	**-**	**-**	**-**
***Volvox*** ^***1*,*3***^	***A B***	**-**	**-**	**-**	**-**	**-**	**-**	**+**	**-**
**Cryptophyta **									
***Chroomonas*** ^***1*,*2*,*3***^	***A B***	**-**	**-**	**-**	**-**	**-**	**-**	**+**	**-**
***Cryptomonas*** ^***1*,*2*,*3***^	***A B C***	**+**	**+**	**-**	**-**	**-**	**-**	**+**	**+**
**Cyanobacteria **									
***Alkalinema*** ^***2***^	***B C D***	**-**	**-**	**+**	**+**	**-**	**-**	**-**	**-**
***Anabaena*** ^***1*,*3***^	***C D***	**+**	**-**	**-**	**-**	**-**	**+**	**-**	**-**
***Aphanizomenon*** ^***1*,*2*,*3***^	***A B C D***	**-**	**-**	**-**	**-**	**+**	**+**	**-**	**-**
***Chroococcus*** ^***1*,*2*,*3***^	***A B C D***	**+**	**-**	**-**	**-**	**-**	**-**	**-**	**-**
***Chrysosporum*** ^***1*,*2*,*3***^	***A B C***	**-**	**-**	**+**	**+**	**-**	**-**	**-**	**-**
***Coelosphaerium*** ^***1*,*2*,*3***^	***A C***	**+**	**-**	**-**	**-**	**-**	**-**	**-**	**-**
***Cyanobium*** ^***1*,*2*,*3***^	***A B C***	**-**	**-**	**+**	**+**	**-**	**-**	**-**	**-**
***Cylindrospermopsis*** ^***1*,*2*,*3***^	***A B C***	**-**	**-**	**+**	**+**	**-**	**-**	**-**	**-**
***Cylindrospermum*** ^***1*,*2*,*3***^	***A B C***	**+**	**-**	**-**	**-**	**+**	**+**	**-**	**-**
***Gloeothece*** ^***1***^	***A***	**-**	**-**	**-**	**-**	**-**	**-**	**+**	**-**
***Leptolyngbya*** ^***1*,*2*,*3***^	***A B C D***	**+**	**-**	**-**	**+**	**-**	**+**	**-**	**-**
***Limnothrix*** ^***1*,*2*,*3***^	***A B C D***	**-**	**-**	**+**	**+**	**-**	**-**	**-**	**-**
***Merismopedia*** ^***1*,*2*,*3***^	***A B C D***	**+**	**-**	**+**	**+**	**+**	**+**	**-**	**-**
***Mirocystis*** ^***1*,*2*,*3***^	***A B C***	**+**	**-**	**+**	**+**	**+**	**+**	**+**	**+**
***Nodosilinea*** ^***3***^	***C***	**-**	**-**	**-**	**+**	**-**	**-**	**-**	**-**
***Nostoc*** ^***1*,*2*,*3***^	***A B C***	**-**	**-**	**-**	**-**	**-**	**-**	**-**	**+**
***Phormidium*** ^***1***^	***C***	**-**	**-**	**+**	**-**	**-**	**-**	**-**	**-**
***Planktothricoides*** ^***1*,*2***^	***A C***	**-**	**-**	**+**	**+**	**+**	**+**	**-**	**-**
***Planktothrix*** ^***2***^	***C***	**-**	**-**	**-**	**-**	**+**	**-**	**-**	**-**
***Prochlorothrix*** ^***1*,*2***^	***A C***	**-**	**-**	**+**	**+**	**+**	**+**	**-**	**-**
***Psuedanabaena*** ^***1*,*2*,*3***^	***A B C D***	**+**	**-**	**+**	**+**	**+**	**+**	**-**	**-**
***Snowella*** ^***1*,*2*,*3***^	***A B C***	**-**	**-**	**+**	**+**	**+**	**+**	**-**	**-**
***Spirulina*** ^***1*,*2*,*3***^	***A B C D***	**+**	**-**	**-**	**-**	**-**	**-**	**-**	**-**
***Synechococcus*** ^***1*,*2*,*3***^	***A B C***	**-**	**-**	**-**	**-**	**+**	**+**	**+**	**+**
**Dinoflagellata **									
***Heterocapsa*** ^***1*,*2*,*3***^	***A B C D***	**-**	**-**	**-**	**-**	**-**	**-**	**-**	**+**
***Thoracosphaera*** ^***1*,*2*,*3***^	***A B C D***	**-**	**-**	**-**	**-**	**-**	**-**	**+**	**+**
**Euglenophyta **									
***Euglena*** ^***1*,*2*,*3***^	***A B C D***	**+**	**-**	**-**	**-**	**-**	**-**	**+**	**+**
**Ochrophyta **									
***Botrydiopsis*** ^***1*,*2*,*3***^	***A B C D***	**+**	**-**	**-**	**-**	**-**	**-**	**-**	**-**

*Genera that were identified through both morphological and molecular level through the four-enrichment media tested: A = BG-11, B = BBM, C = MM, and D = MS. Locations are annotated by number superscribed beside genera name: ^1^Nile1, ^2^Nile2 and ^3^Nile3.

### Taxonomic composition and diversity comparison from different databases

The diversity of the microalgae cultured on the four media was analyzed through two regions of the 16S rRNA gene and one region in the 18S rRNA gene. The two 16S rRNA regions sequenced (V1-V3 and V4-V5) combined had a total of 852,517 sequences. In comparison, the 18S rRNA V4 region had 423,623 sequences. After the filtering and processing, the two 16S rRNA regions combined had 271,511 unique sequences, and the 18S rRNA region had 344,887 sequences. The 16S rRNA OTUs were assigned by alignment to three reference databases: SILVA, Greengenes (GG), and the Protist Ribosomal Reference (PR^2^). The 18S rRNA sequences were aligned to the SILVA database.

5298 taxa were identified using the 16S V1-V3 and SILVA as a reference database, while using the GG and the PR^2^ database, 5498 and 5942 taxa were classified, respectively. Moreover, 397 taxa were classified using the 18S rRNA through the SILVA database. Further filtering was performed to eliminate unclassified phyla to focus on the classified microalgae phyla/genera using RStudio packages.

#### Cyanobacteria identification

**Fig 2:**
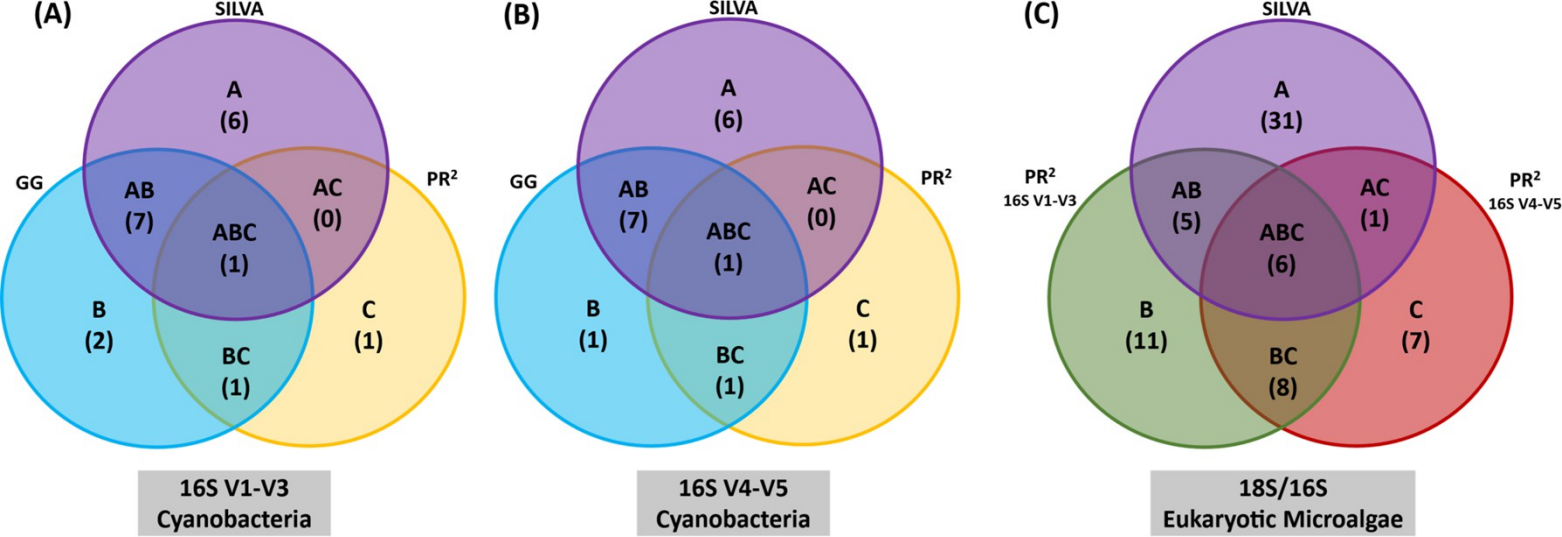
Venn diagrams illustrating the unique and shared microalgae identified through the different databases (SILVA, GG, and PR^2^) and the different DNA barcodes sequenced. Cyanobacteria genera were identified through (A) 16S V1-V3 region and (B) 16S V4-V5 rRNA region. (C) Eukaryotic genera identified through 18S rRNA gene annotated using SILVA database and 16S rRNA gene annotated using PR^2^ database.

**Fig 3:**
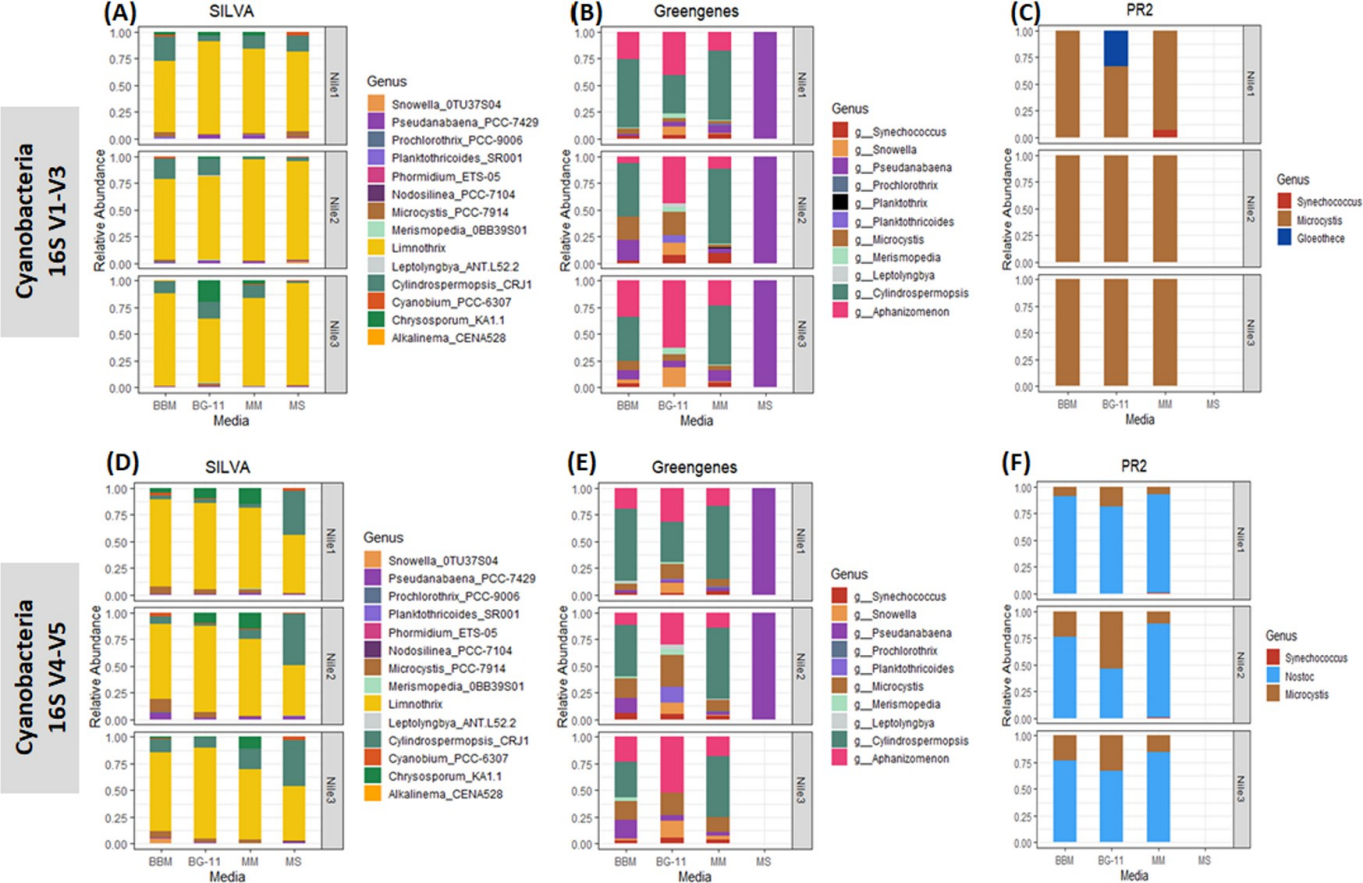
Cyanobacteria identification. Relative abundance of cyanobacteria identified through 16S rDNA V1-V3 (top-half) and 16S rDNA V4-V5 region (bottom-half) annotated through (A & D) SILVA, (B & C) GG and (C & F) PR^2^ databases.

The 16S rRNA hypervariable regions V1-V3, and V4-V5 and three databases identified 18 and 17 genera, respectively **(Fig 2A and 2B)**. By analyzing the two SSU 16S rRNA gene regions sequenced, the SILVA database aided in identifying higher diversity of cyanobacteria (14 genera) over the other tested databases (**Fig 3A and 3D**). Alignment to SILVA alone, both 16S rRNA regions produced identical results, and either region would be sufficient for cyanobacteria identification. Certain cyanobacteria were only identified through GG and PR^2^ databases (**Fig 3B and 3E**). Using the GG database, two genera of cyanobacteria, *Aphanizomenon* and *Planktothrix*, were identified exclusively through the V1-V3 and V4-V5 regions of the 16S rRNA gene and were not identified using SILVA and PR^2^ (**Fig 3**). Not much was expected from the PR^2^ database and identifying cyanobacteria (**Fig 3C and 3F**); however, two cyanobacteria genera were only identified through the PR^2^ database. Using the V1-V3 region, *Gloeothece* was identified in the BG-11 medium, and the V4-V5 region revealed *Nostoc* in the nutrient media BG-11, BBM, and MM. Nonetheless, using the primers V1-V3 and V4-V5 helped to uncover more cyanobacteria using the SILVA and GG databases.

#### Eukaryotic microalgae identification

**Fig 4:**
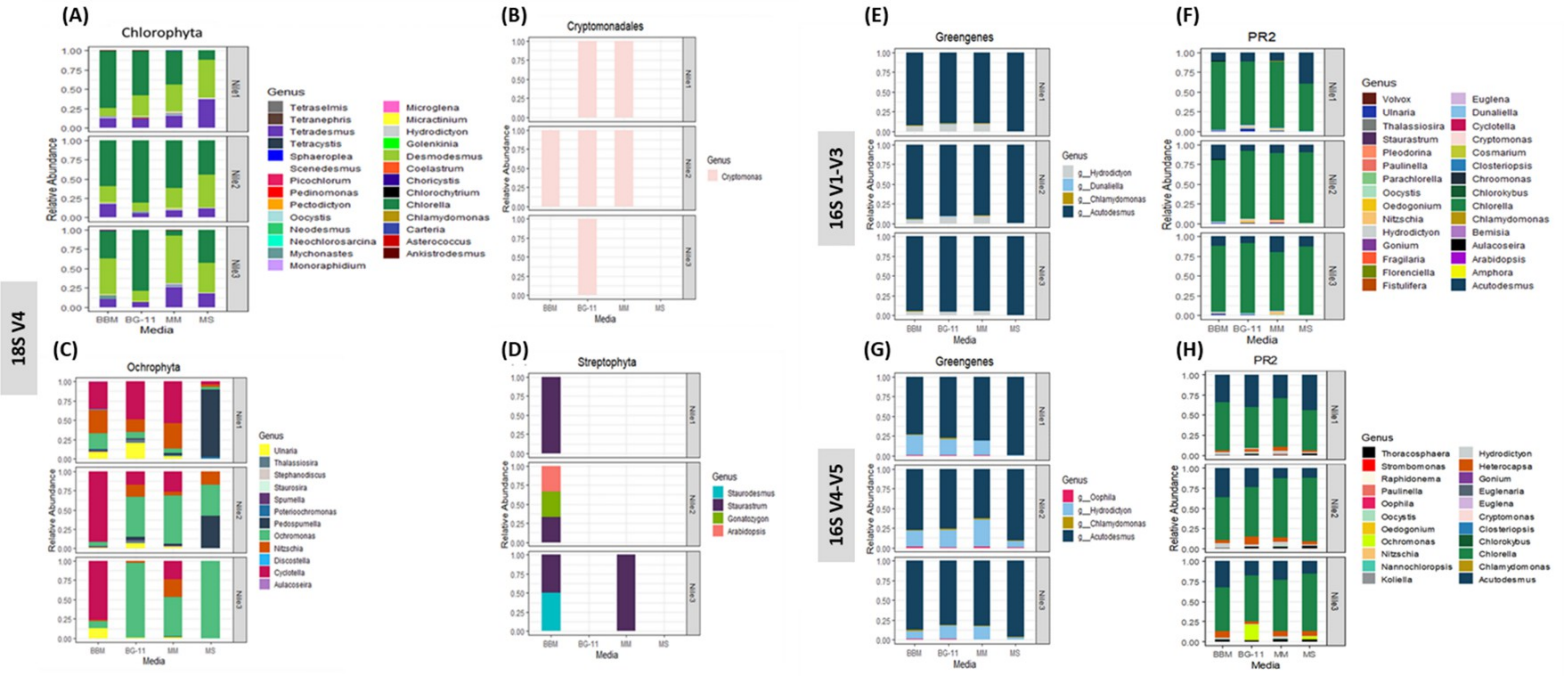
Eukaryotic microalgae identification. Relative abundance of eukaryotic microalgae identified through the different databases (SILVA, GG, and PR^2^) and the different DNA barcodes sequenced. (A-D) are eukaryotic microalgae identified through 18S rRNA gene annotated using SILVA database. Relative abundance of eukaryotic microalgae genera identified using 16S V1-V3 through Greengenes and PR^2^ (E & F), respectively, 16S V4-V5 rRNA gene (G & H) Greengenes and PR^2^, respectively.

Both the 16S and the 18S rRNA sequenced regions identified 68 eukaryotic microalgae (**Fig 2C**). The SILVA database and the 18S rRNA V4 region revealed the highest diversity of 43 eukaryotic microalgae, with 27 genera belonging to the phylum Chlorophyta and 12 genera belonging to the Ochrophyta. The remaining genera belong to the phylum Streptophyta (3 genera) and Cryptophyta (1 genus) (**Fig 4A–4D**). The PR^2^ database and the two 16S rRNA genes classified 37 eukaryotic microalgae genera. The 16S V1-V3 fragment divulged 28 genera, out of which 10 genera were uniquely identified by the PR^2^ database. In contrast, the 16S V4-V5 fragment alignment identified 22 microalgae genera (**Fig 4E–4H**). Using only the SILVA database and the 18S rRNA V4, 25 eukaryotic microalgae would have been missed from the study as these genera were only revealed through the sequencing of the 16S V1-V3 and the V4-V5 regions and annotated using the PR^2^.

### Alpha diversity indices of total microbial community based on OTUs

**Fig 5:**
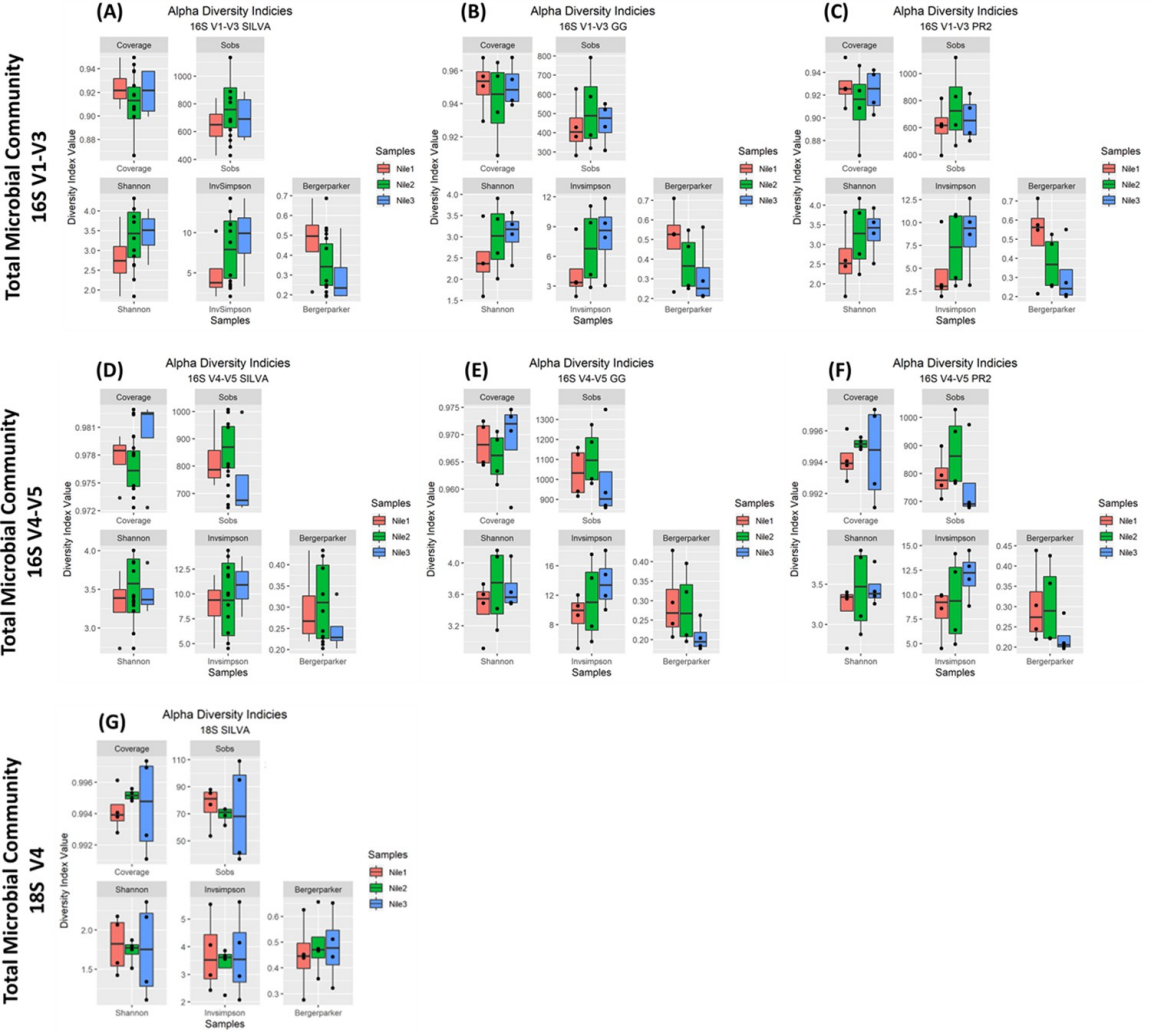
Alpha diversity indices of total microbial community based on OTUs. The total calculated alpha diversity are as follows: Good’s Coverage; S_obs_ (total number of OTUs observed); Shannon; InvSimpson and Breger-Parker. The first half belonging to the V1-V3 region (A-C) and the second-half represents the V4-V5 region (D-F), and last plot belongs to 18S V4. (A, D) SILVA, (B, E) GG, and (C, F) PR^2^ database. (G) Alpha diversity indices calculated through 18S rRNA gene and SILVA.

The enrichment of the microalgae diversity through nutrient media has created a closed ecosystem, and hence the alpha diversity indices are applied to the new community. The diversity indices here are used to validate the enrichment of the microalgae community and do not reflect their natural relative abundance. The 16S V1-V3 region through the three databases had similar Good’s coverage index ranging from 94–95% across the samples using the GG database, while the PR^2^ coverage index ranged from 91–93% (**Fig 5A–5C**). The observed richness (S_obs_) in terms of OTUs was the highest in the Nile2 sample, followed by the Nile3 and Nile1 samples across the three databases (**Fig 5A–5C**). The V4-V5 region of the 16S rDNA had a higher Good’s coverage (<96%) when calculated across the three databases. The S_obs_ are high when analyzed through the V4-V5 region over the V1-V3 region. However, the Nile2 still had the highest S_obs_, followed by Nile1 and Nile3 using both 16S regions (**Fig 5D–5F**). The Shannon index for Nile2 was the highest amongst the other Nile samples when analyzing the sequences for the V4-V5 region (**Fig 5D–5F**), while the higher Shannon index was observed in Nile3 when examining the V1-V3 region (**Fig 5A–5C**), followed by Nile2 and Nile1 samples.

The high InvSimpson index reflects the increase in diversity. The InvSimpson values increase is observed across Nile3 samples across all the sequenced regions, followed by Nile2 and Nile1 samples. However, the 16S V4-V5 and the 18S V4 of Nile1 and Nile2 have a similar median (**Fig 5D–5G**). The final alpha diversity index evaluated regarding the OTUs abundance is the Berger-Parker index. The decrease in this index indicates an increase in diversity. The Nile3 sample has the lowest Berger-Parker index indicating the presence of high diversity followed by Nile2 and Nile3 across all the databases and regions (**Fig 5**). This finding is in line with the other two calculated indices, Shannon and InvSimpson, for the three locations. The 18S V4 region for eukaryotic microorganisms analyzed through the SILVA database had a Good’s coverage >99%, while the highest S_obs_ was observed in the Nile1 sample followed by Nile3 and Nile2. However, Shannon and InvSimpson indices across all the samples were similar Nile1 (1.81, 3.75), Nile2 (1.73, 3.33), and (1.74, 3.69) (Shannon, InvSimpson, respectively) (**Fig 5G**). The Berger-Parker index was also similar for all three samples 0.45, 0.49, and 0.48 for Nile1, Nile2, and Nile3. The diversity indices indicate that the Nile3 is the most abundant and with an even distribution of genera and the absence of the dominance of a genus. This is followed by the Nile2 and Nile1 samples. Regarding the 18S V4 region annotated by SILVA, the diversity indices indicate that Nile1 has the richest diversity with a high evenness between the genera identified. This is followed by Nile3 and then Nile2.

**Fig 6:**
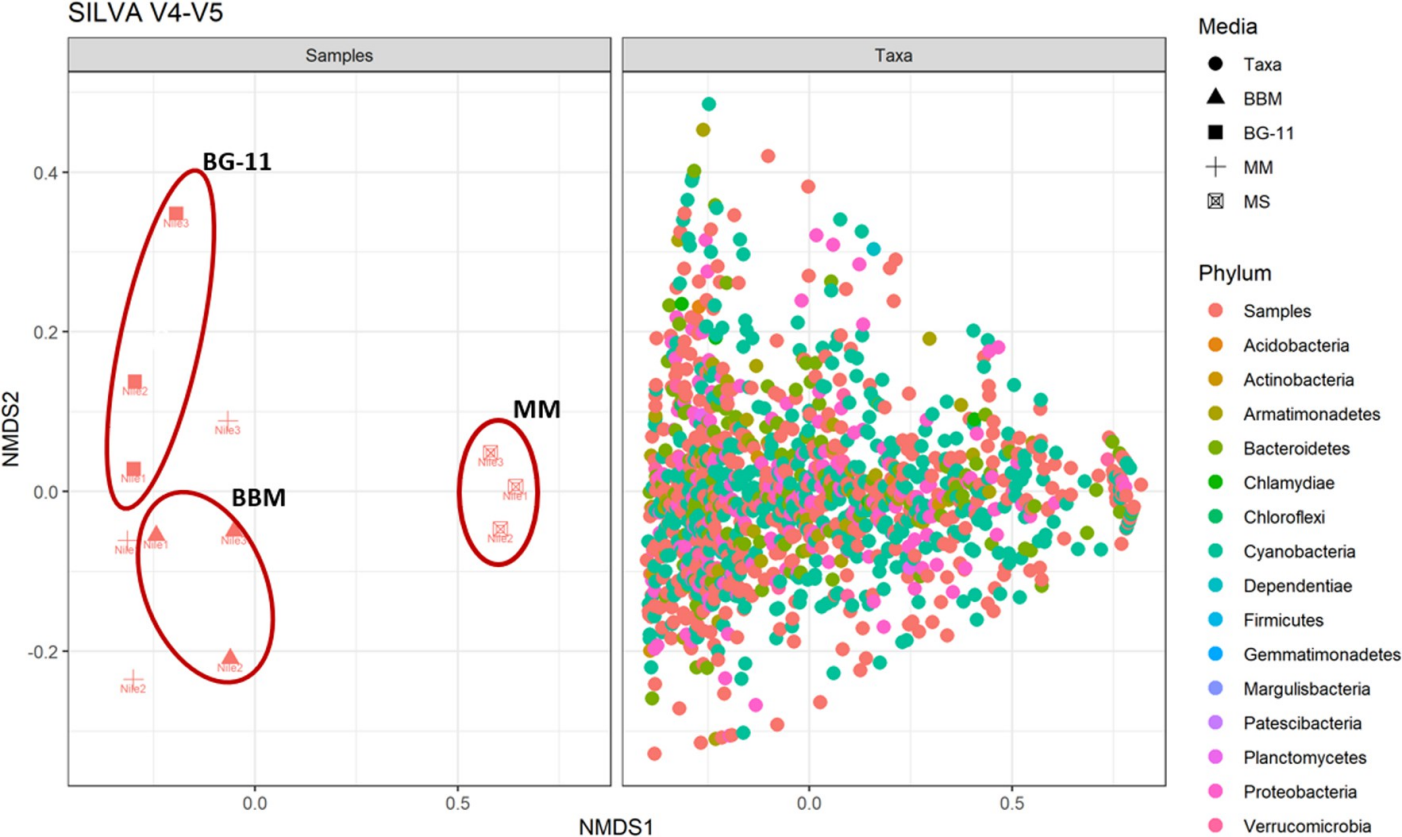
Non-metric multidimensional scaling analysis (nMDS) computed on Bray-Curtis similarity index obtained for microbial community-identified using SILVA 18S V4, water samples, and media as factors.

The relationship between identified phyla, media, and location was examined through the Bray-Curtis similarity index based on the microbial composition identified through the results of the SILVA 18S V4 as it identified the highest overall diversity. An unexpected clustering pattern was observed, and samples were clustered with samples enriched on the same media rather than being clustered by their corresponding locations **(Fig 6)**. This result demonstrates that using only one medium can lead to the enrichment of certain microalgae species while missing others, resulting in misleading data concerning the diversity of studied environmental samples.

## Discussion

This study aimed to evaluate culture-based morphology and the multi-primer/multi-database molecular approach in identifying microalgae diversity from an environmental sample, in our case, the River Nile. The challenge presented was cultivating the highest possible microalgae diversity and identifying them microscopically and through different DNA barcodes. Each of the four nutrient media tested had all the necessary macronutrients (N, P, K, and S) and micronutrients (such as Fe, Cu, Mn, Zn, Co, and Mo) to support microalgae growth. However, each nutrient media contained different macro and micronutrient concentrations. Differences in nutrients and media components could have led to the preference of certain microalgae growth over others, such as nitrogen, which differs among the different media utilized in form and concentration [40–42]. BG-11, BBM, and MM media contain sodium nitrate as the main nitrogen source. The MS medium contains the highest nitrogen concentration in the form of ammonium and potassium nitrate. Microalgae use nitrogen in different forms, such as nitrate, nitrite, ammonium, as inorganic nitrogen sources, and urea, as an organic nitrogen source. The different nitrogen sources are first reduced to ammonium which is converted/integrated into amino acids through different pathways in microalgae [42]. While ammonium is a preferred nitrogen source due to its low-energy assimilation, sodium, and potassium nitrates are still preferred in media over ammonium. This is attributed to ammonium ions (NH_4_^+^) being converted to ammonia gas (NH_3_) under aeration at alkaline conditions and thus considered a loss of nitrogen source [43]. Hence, this may be a reason why MS media had the lowest identified diversity even though it was rich in nitrogen content.

Citric acid and EDTA are other critical media components. The citric acid present in BG-11 and MM media is responsible for solubilizing salt components in the media and preventing iron precipitation, making it readily available to the microalgae, thus resulting in an enhanced growth rate. Iron is considered an essential limiting micronutrient [43]. Therefore, it can be a leading cause of the higher diversity in MM and BG-11 media. BG-11, BBM and MM media also have EDTA, disodium salt acting as a chelating agent. In contrast, MS medium only has its iron chelated in the form of Ferric sodium EDTA, which has been reported to be a better source of iron [44, 45]. Yet, the MS medium did not uniquely enrich any microalgae over the other tested media when morphologically analyzed. This indicated that the ratio of iron is relatively low compared to the other media, and its increase could have resulted in a higher diversity enrichment.

pH can also increase due to the microalgae uptake of inorganic carbon, and photosynthesis increases O_2_ production, which in turn may increase the pH of the culture [46, 47]. In our experiments nutrient content consumption was not measured. Nutrient depletion and accumulation of waste products from the enriched microalgae biomass is assumed after 14 days and can lead to the increase in pH. After 14 days the intermediate and rare microalgae (low in abundance) are further reduced, boosting the dominant and tolerant microalgae such as *Ankistrodesmus*, *Chlorella*, and *Scenedesmus* [48, 49]. This is common as green algae are known to dominate and reduce the growth of cyanobacteria [50].

To perform a molecular-level analysis at time-point zero, it would have been necessary to filter the samples to gather the existing microbiome. However, this filtration process could have caused the loss of picoplankton [51] or dormant cysts, potentially resulting in the omission of certain species [52]. We recognize that conducting molecular analysis on the three samples before enrichment on day zero would have further enhanced our understanding of the algal population dynamics from the beginning. However, filtration of the samples could have resulted in the loss of picoplankton *Choricystis*, *Nannochloropsis*, *Picochlorum*, *Synechococcus*, and *Thalassiosira*. The identification of diversity is significantly influenced by filtration procedures, which involve the selection of filter paper type, pore size, and the amount of environmental sample filtered [53–55]. Given the potential limitations associated with filtration, we decided to proceed with enrichment.

Sixty-seven of 104 microalgae genera were only identified through sequencing and molecular-level identification while only 17 unique genera were identified morphologically. It is possible that the discrepancy is due to the fact that the species had undergone cell lysis either in the environment or during the culturing period during the experiment releasing their genetic material [1] or were in a dormant phase [52]. Accordingly, the DNA of the species has been detected through sequencing and not identified morphologically [56–58]. Moreover, some species may have been missed through microscopy as the method depends on the scientist and their skill in identifying microalgae through morphology [59, 60].

The 17 unique genera identified morphologically were not identified through sequencing. This can be explained by the possibility that some microalgae were incorrectly classified through morphological characteristics due to the close resemblance to other microalgae in the same phylum. For example, *Tetraspora* (Chlorophyta) can be confused with *Chlamydomonas* or *Gloeococcus* while other genera such as *Leptolyngbya* and *Spirulina* (Cyanobacteria) can be incorrectly classified with other filamentous Cyanobacteria [61]. The presence of high nutrients and asexual reproduction can hinder the morphological identification of unicellular microalgae due to their phenotypic plasticity under nutrient and environmental alteration [52, 62].

The number of unique microalgae identified through the molecular-level identification can be higher due to the presence of synonyms of genera between databases that may cause a double count between morphological and molecular-level identification or result in a “hit and miss” between databases [63]. *Aphanizomenon* identified through 16S rRNA primers using GG is considered as a basionym of *Chrysosporum* which was only identified through SILVA using the 16S rRNA primers [64]. *Chrysosporum* has a homotypic synonym *Anabaena bergii* [65, 66] which was identified through microscopy to the genus level as *Anabaena* using MM and MS media in Nile1 but using the two 16S rRNA primer sets and alignment against SILVA database. Other basionym include *Raphidonema* and *Koliella* (Taxonomy ID: 153998 and 33092) [67].

A drawback of the databases used in this study is that they are not specific to microalgae and are not constantly updated with microalgal sequences compared to microbial taxonomies such as in studies on the gut and oral microbiomes or general microbial communities in environmental samples [38, 68–72].

Genera such as *Selenastrum* identified through morphological-level identification has a synonym *Monophidium* profiled through SILVA 18S V4 region. Even though there is a complete sequence entry of the 18S rRNA gene of *Selenastrum* present in the SILVA database along with a sequence for *Monophidium* [73]. This limitation can be ascribed to the SILVA database being a curated database, and there are several entries with different synonyms of the same genera [74]. However, some of the microalgae identified only on a morphological level have characteristic features that can be easily distinguished microscopically and are not mistaken with other genera such as *Actinastrum*, *Coelastrum*, *Dictyosphaerium*, *Largerheimia*, *Tetraedron*, *Pediastrum*, and *Selenastrum* [31, 32]. Nevertheless, these species were not identified on a molecular level through any of the regions analyzed or through the three reference databases used. The complete 18S rRNA gene of the genera *Actinastrum*, *Coelastrum*, *Dictyosphaerium*, *Tetraedron*, *Pediastrum*, and *Selenastrum* are present in the SILVA database, while *Largerheimia* 18S rRNA gene was not available on SILVA but is present in a different database ENA (European Nucleotide Archive) that has not been used in this study for the analysis of the 18S rRNA sequences [73].

BG-11, BBM and MM were the most suitable for culturing and enrichment of the highest diversity from an environmental sample, compared to MS media that did not identify any unique species. The enrichment using culture-based methods can be considered an alternative to the more expensive approach of increasing the number of sequence reads to detect the less abundant species due to limited detection thresholds. The culture-based method followed by the molecular level using several different hypervariable regions eliminates these biases resulting from using one medium or a sequencing region and broadens the identification level. It is critical to recognize that different enrichment media and sequencing regions complements one another and provides a more complete diversity profile.

The combined use of both the culture-based method and sequence analysis of multiple rRNA regions/genes (targeting both nuclear and plastid genomes) [75, 76] facilitates a more reliable and comprehensive approach to identifying total microalgae biodiversity. This approach can be considered promising as it targets microalgae identification from an environmental sample compared to the commonly used metagenomic sequencing that utilizes the environmental sample directly. These are usually high in microbial biomass and low in microalgae biomass. We acknowledge that the culturomics approach to identifying microalgae from environmental studies needs further improvement and further studies are needed to improve the method and further define it for reproducibility. This approach to identifying microalgae can be further explored using samples from different ecosystems.

## Conclusion

Four enrichment media were employed in this study to examine their efficacy in enhancing the diversity of microalgae obtained from the environment. The two 16S rDNA regions and one 18S rDNA region were then tested and aligned to various databases to determine the best alternative methods for identifying microalgae to classical microscopy. Overall, we advise using both morphological and morphological approaches since dependence on one technique over another can be restrictive and result in genera omission. When relying on molecular-level identification, the reference database and the DNA barcode employed must be considered from several prospective. The SILVA database was the most effective in identifying cyanobacteria and both 16S rDNA regions produced identical results. Whereas, Greengenes database identified less cyanobacteria genera compared to SILVA using the 16S rDNA Most of the detected eukaryotic microalgae could be recognized mainly using the 18S V4 region and alignment to SILVA, followed by the alignment of the 16S V1–V3 to the PR2. Therefore, we recommend coupling enrichment media (BG-11, BBM, and MM) with morphological level identification, followed by molecular level identification using the 16S V1-V3 or V4-V5 region and 18S V4 region for both cyanobacteria and eukaryotic microalgae identification.

## Supporting information

S1 Fig Growth curve across all tested media to ensure enrichment and media suitability uniform algal growth. (TIF)

S1 Table The geographic coordinate for environmental samples collected from the river Nile and used in this study. (PDF)

S2 Table The chemical composition (mg L^-1^) for the four growth media used in the study. (PDF)

S3 Table Physiochemical analysis of collected samples. (PDF)

S4 Table Primer sequences used for PCR amplification and sequencing. (PDF)

S5 Table Unique and shared genera identified across the four different media A = BG-11, B = BBM, C = MM and D = MS. (a) morphologically across the four media, (b) molecularly using the three primer- sets and the three reference databases, and (c) combining both molecular and morphological approaches.
(PDF)
