# Supplementary material for: Comparative study of multiple approaches for identifying cultivable microalgae population diversity from freshwater samples
Source: PLoS One. 2023 Jul 7;18(7):e0285913. doi: 10.1371/journal.pone.0285913 (PMC10328328; doi:10.1371/journal.pone.0285913)
Supplement: S1 Table — (PDF) [file pone.0285913.s002.pdf]

S1 Table. The geographic coordinate for environmental samples collected from the river Nile and used in this study.

Location Code	Geographical coordinates
Nile1	29°59'51.6"N; 31°12'55.6"E
Nile2	30°00'58.0"N; 31°13'05.4"E
Nile3	30°00'55.4"N; 31°13'41.8"E
