# Supplementary material for: Comparative study of multiple approaches for identifying cultivable microalgae population diversity from freshwater samples
Source: PLoS One. 2023 Jul 7;18(7):e0285913. doi: 10.1371/journal.pone.0285913 (PMC10328328; doi:10.1371/journal.pone.0285913)
Supplement: S2 Table — (PDF) [file pone.0285913.s003.pdf]

S2 Table. The chemical composition (mg L^{-1}) for the four-growth media used in the study.

Nutrients	BG-11	BBM	MM	MS
Ammonium Nitrate	-	-	-	825
Boric Acid	2.86	11.42	7.14	3.1
Calcium Chloride, Anhydrous	27.18	18.87	23.025	166.5
Citric Acid, Anhydrous	6	-	3	-
Cobalt Nitrate·$6H_2O$	0.049	0.49	0.2695	-
Cobalt Chloride·$6H_2O$	-	-	-	0.0125
Cupric Sulfate·$5H_2O$	0.079	1.57	0.8245	0.0125
EDTA, Disodium Salt	1	63.61	32.305	-
Ferric Ammonium Citrate	6	-	3	-
Ferric Sodium EDTA	-	-	-	18.35
Ferrous Sulfate·$7H_2O$	-	4.98	2.49	-
Magnesium Sulfate·$7H_2O$	75	-	55.815	-
Magnesium Sulfate, Anhydrous		36.63		90.5
Manganese Chloride·$4H_2O$	1.81	1.44	1.625	-
Manganese Sulfate·H_2O	-	-	-	8.45
Molybdic Acid (Sodium Salt) ·$2H_2O$	-	-	-	0.125
Potassium Hydroxide	-	31	15.5	-
Potassium Iodide	-	-	-	0.415
Potassium Phosphate, Dibasic	40	75	57.5	-
Potassium Phosphate, Monobasic	-	175	87.5	85
Sodium Carbonate Anhydrous	20	-	10	-
Sodium Chloride	-	25	12.5	-
Sodium Molybdate·$2H_2O$	0.39	1.19	0.79	-
Sodium Nitrate	1500	250	875	-
Potassium Nitrate	-	-	-	950
Zinc Sulfate· $7H_2O$	0.222	8.82	4.521	4.3
