# Supplementary material for: Comparative study of multiple approaches for identifying cultivable microalgae population diversity from freshwater samples
Source: PLoS One. 2023 Jul 7;18(7):e0285913. doi: 10.1371/journal.pone.0285913 (PMC10328328; doi:10.1371/journal.pone.0285913)
Supplement: S3 Table — (PDF) [file pone.0285913.s004.pdf]

S3 Table. Physicochemical analysis of collected samples.

Physicochemical Parameters	Nile1	Nile2	Nile3
pH	7.50	7.50	7.20
Electrical Conductivity (µS/cm)	600	590	600
Total Dissolved Solids (ppm)	382.0	375.0	382.0
Chemical Parameters (mg $L^{-`}$)			
HCO_3^-	103.7	92.1	109.2
Cl^-	103.9	89.7	114.9
SO_4^{-2}	96.1	94.6	73.5
Ca^{2+}	39.5	39.5	33.8
Mg^{2+}	32.8	28.1	34.8
Na^+	41.6	43.2	43.2
K^+	5.9	5.08	5.47
NO_3^-	5.53	5.95	6.3
NH_4^+	3.15	1.82	1.96
Iron	0.014	0.053	0.062
Phosphorus	<1.5 †	<1.5 †	<1.5 †
Manganese	0.028	0.019	0.022
Zinc	0.033	<0.2 †	<0.2 †
Copper	<0.2 †	<0.2 †	<0.2 †
Boron	0.004	<0.3 †	0.003

† Unit (µg L^{-1})
