# Supplementary material for: Comparative study of multiple approaches for identifying cultivable microalgae population diversity from freshwater samples
Source: PLoS One. 2023 Jul 7;18(7):e0285913. doi: 10.1371/journal.pone.0285913 (PMC10328328; doi:10.1371/journal.pone.0285913)
Supplement: S4 Table — (PDF) [file pone.0285913.s005.pdf]

S4 Table. Primer sequences used for PCR amplification and sequencing.

Amplified Gene	Primer	Primer sequences (5′-3′)	References
16S V1-V3	8F	AGAGTTTGATYMTGGCTCAG	(Turner et al., 1999; Watts et al., 2017)
	518R	ATTACCGCGGCTGCTGG	
16S V4-V5	515F	GTGCCAGCMGCCGCGG	(Turner et al., 1999)
	907R	AATCCRAGAATTTCACCTCT	
18S V4	528F	GCGGTAATTCCAGCTCCAA	(Heidelberg et al., 2013; Wolf, Kilias, & Metfies, 2014)
	706R	AATCCRAGAATTTCACCTCT	
