# Supplementary material for: Comparative study of multiple approaches for identifying cultivable microalgae population diversity from freshwater samples
Source: PLoS One. 2023 Jul 7;18(7):e0285913. doi: 10.1371/journal.pone.0285913 (PMC10328328; doi:10.1371/journal.pone.0285913)
Supplement: S5 Table — (a) morphologically across the four media, (b) molecularly using the three primer- sets and the three reference databases, and (c) combining both molecular and morphological approaches. (PDF) [file pone.0285913.s006.pdf]

S5 Table. Unique and shared genera identified across the four different media A= BG-11, B=BBM, C=MM and D=MS.

(a) Genera identified using morphological level identification across the four-enrichment media.

Number of Genera per set →	1	1	5	19	6	1	4
	A	B	C	ABCD	ABC	BC	AC
Genera	*Staurastrum*	*Cosmarium*	*Mougeotia*	*Anctinastrum*	*Botryococcus*	*Anabaena*	*Chlamydamonas*
			Chlorococcum	*Ankistrodesmus*	*Crucigenia*		*Golenkinia*
			Gonium	*Botrydiopsis*	*Pediastrum*		*Coelosphaerium*
			Cryptomonas	*Chlorella*	*Cylindrospermum*		*Euglena*
			Microcystis	*Chroococcus*	*Merismopedia*		
				Coelastrum	*Psuedanabaena*		
				Cyclotella			
				Dictyosphaerium			
				Lagerheimia			
				Leptolyngbya			
				Micractinium			
				Nitzschia			
				Oocystis			
				Scenedesmus			
				Selenastrum			
				Spirulina			
				Tetraedron			
				Tetraselmis			
				Tetraspora			

(b) Genera identified using molecular-level identification across the four-enrichment media.

Number of Genera per set →	7	3	9	1	37		18
	A	B	C	D	ABCD		ABC
Genera	*Amphora*	*Gonatozygon*	*Staurosira*	*Raphidonema*	*Cyclotella*	*Tetradesmus*	*Aulacoseira*
	Fistulifera	*Staurodesmus*	*Stephanodiscus*		*Nitzschia*	*Heterocapsa*	*Discostella*

	Cosmarium	*Tetraselmis*	*Asterococcus*		*Ochromonas*	*Thoracosphaera*	*Florenciella*
	Golenkinia		*Coelastrum*		*Pedospumella*	*Euglena*	*Nannochloropsis*
	Pectodictyon		*Tetranephris*		*Ulnaria*	*Aphanizomenon*	*Spumella*
	Gloeothece		*Nodosilinea*		*Acutodesmus*	*Chrysosporum*	*Thalassiosira*
	Stromobomonas		*Phormidium*		*Ankistrodesmus*	*Cyanobium*	*Chlorokybus*
			Planktothrix		*Carteria*	*Cylindrospermopsis*	*Staurastrum*
			Euglenaria		*Chlamydamonas*	*Limnothrix*	*Dunaliella*
					Chlorella	*Merismopedia*	*Mychonastes*
					Choricystis	*Psuedanabaena*	*Pedinomonas*
					Closteriopsis	*Sphaeroplea*	*Cryptomonas*
					Desmodesmus	*Neodesmus*	*Leptolyngbya*
					Gonium	*Oocystis*	*Mirocystis*
					Hydrodictyon	*Oophila*	*Nostoc*
					Koliella	*Parachlorella*	*Snowella*
					Micractinium	*Picochlorum*	*Synechococcus*
					Microglena	*Scenedesmus*	*Paulinella*
					Monoraphidium		

(b) Continued:

Number of Genera per set →	1	4	1	3	2	1
	ABD	BCD	ACD	AB	AC	BD
Genera	*Tetracystis*	*Poterioochromonas*	*Fragilaria*	*Oedogonium*	*Planktothricoides*	*Chlorochytrium*
		Neochlorosarcina		*Volvox*	*Prochlorothrix*	
		Pleodorina		*Chroomonas*		
		Alkalinema				

(c) Genera identified using the two-approaches morphological and molecular combined.

Number of Genera per set →	20	17	67		
	AB	A	B		
Genera	*Ankistrodesmus*	*Anabaena*	*Acutodesmus*	*Heterocapsa*	*Pleodorina*
	Chlamydamonas	*Anctinastrum*	*Alkalinema*	*Hydrodictyon*	*Poterioochromonas*
	Chlorella	*Botrydiopsis*	*Amphora*	*Koliella*	*Prochlorothrix*

	Coelastrum	*Botryococcus*	*Aphanizomenon*	*Limnothrix*	*Raphidonema*
	Cosmarium	*Chlorococcum*	*Asterococcus*	*Microglena*	*Snowella*
	Cryptomonas	*Chroococcus*	*Aulacoseira*	*Monoraphidium*	*Sphaeroplea*
	Cyclotella	*Coelosphaerium*	*Carteria*	*Mychonastes*	*Spumella*
	Euglena	*Crucigenia*	*Chlorochytrium*	*Nannochloropsis*	*Staurodesmus*
	Golenkinia	*Cylindrospermum*	*Chlorokybus*	*Neochlorosarcina*	*Staurosira*
	Gonium	*Dictyosphaerium*	*Choricystis*	*Neodesmus*	*Stephanodiscus*
	Leptolyngbya	*Lagerheimia*	*Chroomonas*	*Nodosilinea*	*strombomonas*
	Merismopedia	*Mougeotia*	*Chrysosporum*	*Nostoc*	*Synechococcus*
	Micractinium	*Pediastrum*	*Closteriopsis*	*Ochromonas*	*Tetracystis*
	Mirocystis	*Selenastrum*	*Cyanobium*	*Oedogonium*	*Tetradesmus*
	Nitzschia	*Spirulina*	*Cylindrospermopsis*	*Oophila*	*Tetranephris*
	Oocystis	*Tetraedron*	*Desmodesmus*	*Parachlorella*	*Thalassiosira*
	Psuedanabaena	*Tetraspora*	*Discostella*	*Paulinella*	*Thoracosphaera*
	Scenedesmus		*Dunaliella*	*Pectodictyon*	*Ulnaria*
	Staurastrum		*EUGLENARIA*	*Pedinomonas*	*Volvox*
	Tetraselmis		*Fistulifera*	*Pedospumella*	
			Florenciella	*Phormidium*	
			Fragilaria	*Picochlorum*	
			Gloeothece	*Planktothricoides*	
			Gonatozygon	*Planktothrix*	
